# Tumor-derived exosomes: immune properties and clinical application in lung cancer

**DOI:** 10.20517/cdr.2021.99

**Published:** 2022-02-08

**Authors:** Jing Wu, Suyao Li, Pengfei Zhang

**Affiliations:** Department of Medical Oncology, Zhongshan Hospital, Fudan University, Shanghai 200032, China.

**Keywords:** Lung cancer, tumor-derived exosomes, cancer biomarkers, immunotherapies

## Abstract

Lung cancer is the leading cause of cancer-related death worldwide. Despite advances in diagnosis and treatment of lung cancer, the overall survival remains poor. Evidence indicates that lung cancer development is a complex and dynamic process that involves interactions between tumor cells and their microenvironments, including immune cells. Exosomes are small extracellular vesicles secreted by most cell types; they contain functional molecules that allow intercellular communication. Tumor-derived exosomes (TEXs) carry both immunosuppressive and immunostimulatory mediators and may be involved in various immunomodulatory effects. TEXs, which partially mimic profiles of the parent cells, are a potential source of cancer biomarkers for prognosis, diagnosis, and prediction of response to therapy. In addition, TEXs may interfere with immunotherapies, but they also could be used as adjuvants and antigenic components in vaccines against lung cancer. In the context of lung cancer, identifying TEXs and understanding their contribution to tumorigenesis and the response to immunotherapies represents a challenging research area.

## INTRODUCTION

Lung cancer is one of the most common malignant tumors with the highest morbidity and mortality worldwide^[1]^. Recently, immunotherapies have shown more effectiveness than traditional chemotherapy, and they have dramatically changed the treatment paradigm for lung cancer^[2-4]^. However, only a small proportion of patients can benefit from the immunotherapies, with primary and secondary resistance complicating treatment. One potential explanation for this phenomenon is the complexity and diversity of the tumor microenvironment (TME). Interactions of lung cancer cells with the surrounding TME are critical to cancer progression and response to immunotherapy. In recent years, the communication mediated by exosomes has extensively gained attention.

Exosomes are small bilayer membrane vesicles with a size of 30-100 nm in diameter that are secreted by various cell types such as tumor cells, immune cells, and fibroblasts^[5,6]^. Exosomes derived from tumor cells are referred to as tumor-derived exosomes (TEXs)^[7]^. TEXs have been shown to contain a variety of biomolecules including nucleic acids, proteins, enzymes, and lipids, which are involved in cancer progression, intercellular communication, and immunological function^[8]^. Studies have increasingly indicated that the number and composition of TEXs can in part reflect their cells of origin and biological state, which may serve as potential biomarkers in diagnosis and prognosis of cancer^[9-11]^. TEXs may affect cancer immunotherapy either by sequestration of therapeutic antibodies or supplying self-antigen carriers to improve cancer vaccine efficacy. Their biological roles in cancer progression as well as cancer immunotherapy and biomarkers have indicated that TEXs are critical components of the TME.

In this review, we first describe how TEXs are formed and released to the extracellular matrix, and discuss the composition of TEXs. Then, we outline the immunomodulatory function of TEXs in the lung cancer microenvironment. Moreover, we focus on the utility of TEXs as diagnostic and prognostic biomarkers in lung cancer. Finally, the recent findings on TEXs in immunological changes during immunotherapy are discussed.

## THE FORMATION, RELEASE, AND COMPOSITION OF TEXS

**Figure 1:**
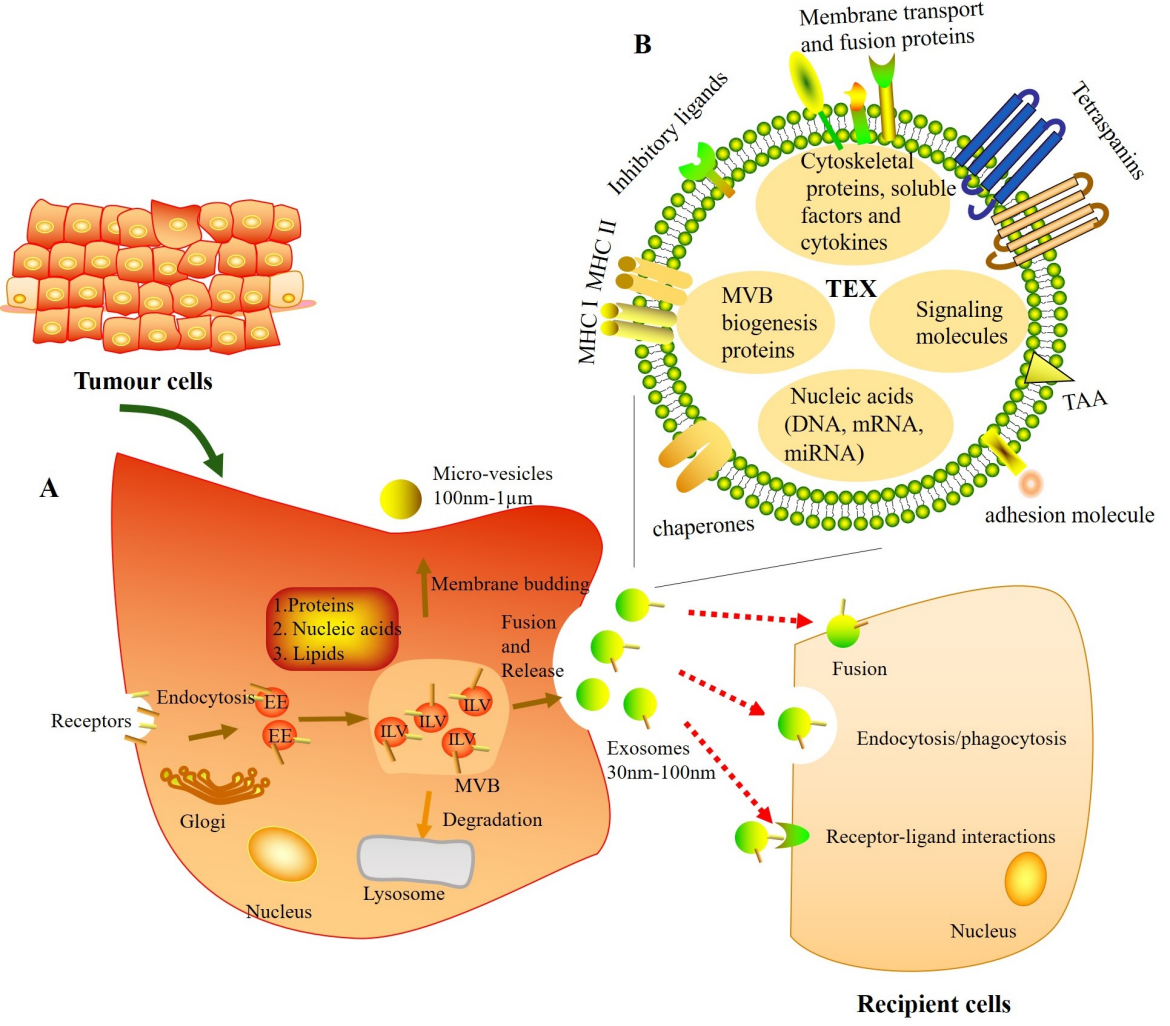
Molecular composition, biogenesis, release, and uptake of tumor-derived exosomes (TEXs). (A) TEXs originate from intraluminal vesicles (ILVs) in the multivesicular bodies (MVBs) (also known as late endosomes). Firstly, early endosomes (EEs) are formed when the membrane microdomains are endocytosed via inward budding of the plasma membrane. Then EEs mature into MVBs, which follow either fusion with the plasma membrane to form exosomes or degradation by lysosome. During this process, the proteins, nucleic acids, and lipids are packed into exosomes. Finally, exosomes can interact with recipient cells through three main ways: endocytosis/phagocytosis, direct fusion with cellular membrane, and receptor-ligand interactions. (B) Schematic diagram of components of TEXs.

TEXs, released by tumor cells, are present ubiquitously in tumor tissues and body fluids^[12]^. Exosome biogenesis initiates from the production of early endosomes via the internalization of membrane microdomains. The limiting membrane of early endosomes bud inwardly to form intraluminal vesicles, then becoming the multivesicular bodies. Finally, exosomes are released when multivesicular bodies fuse with the plasma membrane^[13]^. This formation of exosomes is a tightly regulated process; it involves two pathways through an Endosomal Sorting Complex Required for Transport (ESCRT)-dependent machinery or an ESCRT-independent machinery^[14]^. Once exosomes released, they are able to transfer information to their recipient cells through three main ways: endocytosis/phagocytosis, direct fusion with cellular membrane, and receptor-ligand interactions^[13] ^[Figure 1A].

TEXs consist of a lipid-protein bilayer membrane, including membrane transport and fusion proteins (e.g., annexins, Rab proteins, and flotillin), MHC (class I and II molecules), adhesion molecules (e.g., ICAM, EPCAM, CD44, and integrins), inhibitory ligands [e.g., FasL, TRAIL, PD-L1, and transforming growth factor (TGF)-β/LAP], tetraspanins (e.g., CD9, CD63, CD81, and CD82), tumor associated antigens, chaperones [e.g., heat-shock protein (HSP) 70 and HSP90], lipids, and glycolipids^[12,13,15-17]^. In their lumen, TEXs carry a variety of multivesicular bodies biogenesis proteins [e.g., Alix and tumor susceptibility gene 101 (TSG101)], cytoskeletal proteins (e.g., actin, tubulin, and vimentin), histones, oncoproteins, soluble factors, enzymes, cytokines, signaling molecules, and nucleic acids (including DNA, mRNA, and miRNA)^[13,15] ^[Figure 1B]. The TEXs molecular and genetic content mimics that of parent cells, and is in part considered as surrogates of the parent tumor cells^[18]^. Moreover, TEXs can transfer messages from the parent tumor to recipient cells, including immune cells, within the TME^[19]^.

## EFFECT OF TEXS FOR IMMUNE REGULATION IN LUNG CANCER

**Figure 2:**
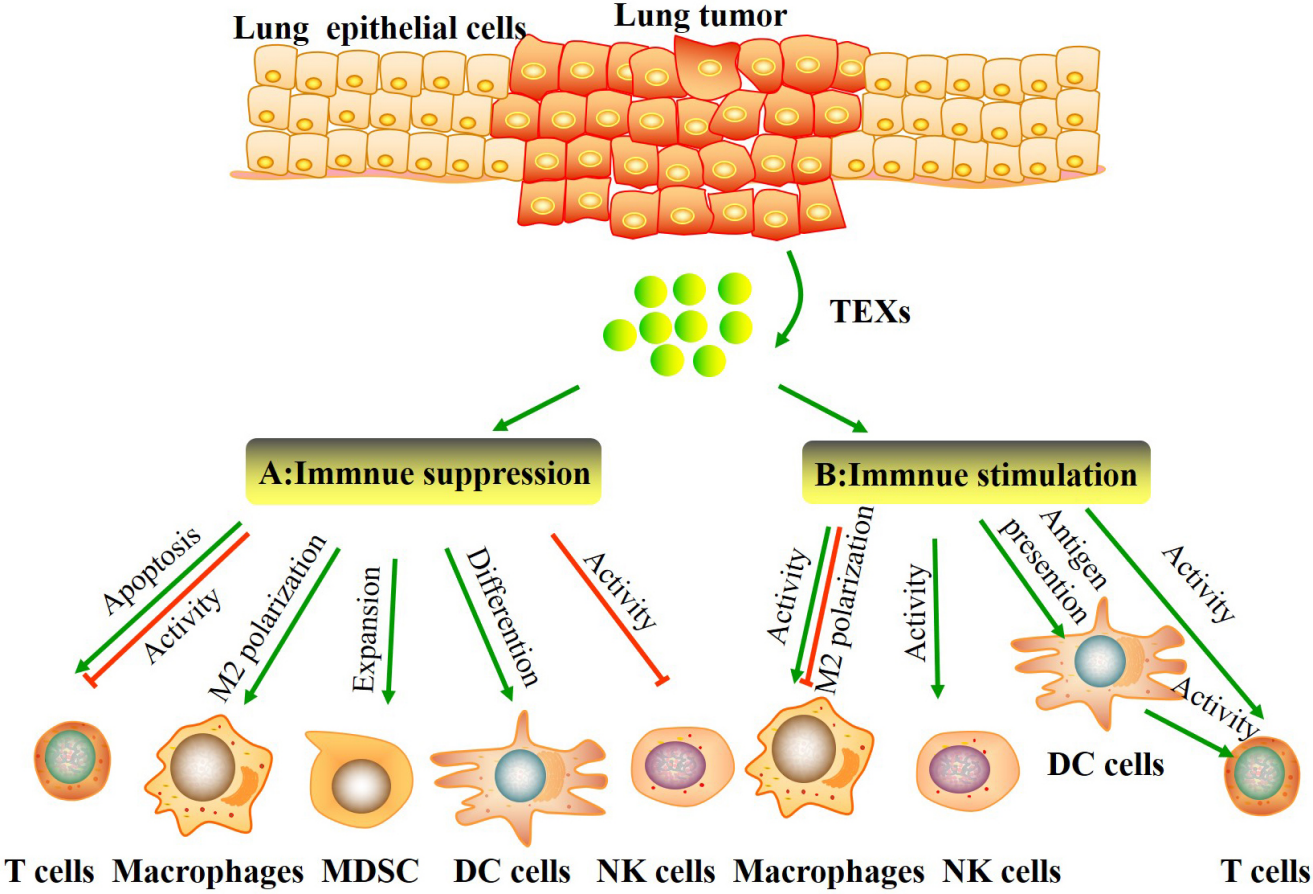
Tumor-derived exosomes (TEXs) carry and deliver both immunosuppressive and immunostimulatory signals to immune cells in the lung tumor microenvironment. (A) Immune suppression. TEXs contribute to establish an immunosuppressive TME by inducing apoptosis and inhibiting the activity of effector T cells, skewing M2 polarization of macrophages, expanding myeloid-derived suppressor cells (MDSCs), suppressing DCs differentiation, and impairing the function of NK cells. (B) Immune stimulation. TEXs can also stimulate immune cells to support antitumor activities, including enhancing the activity of macrophages and NK cells, suppressing M2 macrophage polarization, and increase T cells activity directly or indirectly.

Recently, TEXs have been proposed to act as crucial mediators between cellular communication by transferring both immunosuppressive and immunostimulatory signals to immune cells in a lung cancer microenvironment^[20,21] ^[Table 1, Figure 2].

**Table 1:** Overview of exosomal cargo, source of exosomes, and their biological effects

**Exosomal cargo**	**Donor**	**Biological effect**	**Ref.**
PD-L1	H1299, H358, and H1264	Inactivate T cells	[23]
EGFR	NSCLC biopsies	Induce tolerogenic DCs	[28]
miR-214	Lewis lung carcinoma cells	Downregulate the PTEN-mediated signaling	[29]
miR-433	Plasma of NSCLC patients	Inactivate the WNT/β‑catenin signaling	[31]
miR-21/29a	A-549 and SK-MES	Activate TLR7 and TLR8	[32]
miR-21a	Lewis lung carcinoma cells	Promote MDSCs expansion	[51]
miR-103a	CL1-5 lung cancer cells	Activate of PI3K/Akt and STAT3 signaling pathways	[52]
circFARSA	A549 and PC9 cells	Polarize macrophages to the M2 phenotype	[54]
miR-770	A549 cells	Suppress M2 macrophage polarization	[62]

NSCLC: Non‑small cell lung cancer; DCs: dendritic cells; MDSCs: myeloid-derived suppressor cells.

### Immunosuppressive effect of TEXs

TEXs modulate the activity of T cells by promoting apoptosis and inhibiting proliferation of CD8^+^ T cells^[22]^. Recent studies have indicated that TEXs contain PD-L1, which inhibits T cells activity and promotes tumor progression. Exosomes from lung cancer, melanoma, and breast cancer carry PD-L1 on their surface, which interacts with PD-1 via the extracellular domain, and thereby inactivate T cells^[23]^. Poggio *et al*.^[24]^ discovered that the majority of PD-L1 could be presented on the surface of TEXs, and genetic blockade of exosomal PD-L1 could activate an anti-tumor immune response leading to extend survival in a subset of cancer patients. Moreover, it is suggested that TEXs with FasL expression could induce CD8^+^ T cell apoptosis^[25,26]^. Czystowska *et al*.^[27] ^uncovered the mechanism that the PI3K/Akt pathway was a central target for TEXs in regulating CD8^+^ T cell apoptosis. Huang *et al*.^[28] ^indicated that about 80% of exosomes isolated from non-small cell lung cancer (NSCLC) biopsies contained EGFR. These exosomes can be captured by dendritic cells (DCs). Then tolerogenic DCs were generated and induced tumor antigen specific regulatory T cells (Tregs), which could inhibit the function of tumor specific CD8^+^ T cells. Yin *et al*.^[29]^ found that miR-214 was delivered into recipient CD4^+^ T cells via TEXs, so as to downregulate the PTEN-mediated signaling, thereby promoting Treg expansion and tumor growth. Interestingly, co-incubation of Treg with TEXs may enhance Treg number as well as its suppressive function with the increased production of inhibitory cytokines, TGF-β1 and interleukin (IL)-10^[30]^. Additionally, Liu *et al*.^[31]^ showed that exosome-derived miR-433 inactivated the WNT/β-catenin signaling pathway via targeting transmembrane p24 trafficking protein 5, thus increasing infiltration of CD4^+^ and CD8^+^ cells in NSCLC.

Exosome-derived miR-21/29a derived from A-549 and SK-MES cells promoted lung cancer growth and metastasis through activating Toll like receptors TLR7 and TLR8 on immune cells including NK cells^[32]^. NK cells express a variety of receptors that are either stimulatory or inhibitory^[33]^. The downregulation of those stimulatory receptors, particularly NKG2D, may play an important role in decreasing activity of NK cells in lung cancer patients^[34]^. TEXs originating from hypoxic tumor cells deliver TGF-β1 to NK cells, and thereby reduce NKG2D expression resulting in lower activity of NK cells^[35]^. TEXs can also attenuate NK cell activity via multiple mechanisms including shedding the NKG2D ligand on tumor cells, suppressing Janus kinase (Jak) 3 activation, inhibiting perforin or cyclin D3 production and down-regulation of IL-2-mediated pathways^[36-41]^. Moreover, TEX-carried MICA and MICB ligands can downregulate the stimulatory receptors, especially NKG2D on NK cells^[42]^.

TEXs suppressed the functioning of the immune system by affecting the monocyte differentiation and maturation^[43,44]^. TEXs were capable of blocking DCs migration to lymph nodes through inhibiting most C-C/C-X-C chemokine receptor expression^[45]^. Co-incubation of DCs with TEXs leaded to the down regulation of CD80 and CD86, and subsequently inhibited DCs maturation^[46]^. In addition, TEXs have been shown to induce the differentiation of myeloid precursor cells into highly suppressive myeloid-derived suppressor cells (MDSCs)^[39,47]^. Heat-shock protein 72 (Hsp72) on the TEXs surface could trigger the STAT3 activation and autocrine IL-6 production in MDSCs via a TLR2/MyD88-dependent manner, leading to the increment of immunosuppressive activity of MDSCs^[48-50]^. Zhang *et al*.^[51]^ indicated that miR-21a enriched in lung carcinoma cell-derived exosomes could promote MDSCs expansion via targeting PDCD4, thus enhancing tumor growth. Hsu *et al*.^[52]^ reported that miR-103a was upregulated in lung cancer derived exosomes under hypoxic conditions. Exosomal miR-103a could cause the activation of PI3K/Akt and STAT3 signaling pathways by directly targeting PTEN, thereby resulting in the enhancement of M2 polarization^[52]^. Additionally, the study with A549 (wild-type p53 allele) and H358 (p53 null allele) exosomes suggested that lung cancer cell-derived exosomes mediated M2 polarization may be p53 independent^[53]^. Another study showed that exosomal circFARSA was significantly upregulated in NSCLC tissues and stimulated NSCLC cell metastasis by polarizing macrophages to the M2 phenotype^[54]^.

Taken together, these data suggested that TEXs can modulate the immune response by transferring immunosuppressive signals to immune cells, which in turn contribute to tumor progression^[7,34,55,56]^.

### Immunostimulatory effect of TEXs

TEXs have been reported to be involved in the suppression of the immune system in previous studies. However, since TEXs also carry stimulatory molecules that contribute to activating immune responses, recent studies also focused on anti-tumor immunity of exosomes.

TEXs can act as presenters participating in direct and indirect antigen presentation^[57]^. As direct presenters, TEXs present antigen to T cells via an MHC-peptide complex on their surface. On the other hand, TEXs can also indirectly transfer tumor antigen to antigen presenting cells, like DCs, and then activate the cytotoxic activity of CD8^+^ T cells and CD4^+^ T helper cells, so as to inhibit tumor growth^[58]^. Additionally, the enrichment of HSPs on TEXs such as Hsp70, can stimulate the activity of NK cells^[59]^ and macrophages^[60]^, and induce MHC class I-restricted cytotoxic T cells activation^[61]^. Tumor cell-derived exosomal miR-770 could suppress M2 macrophage polarization via targeting MAP3K1, which in turn decreased NSCLC tumor growth^[62]^. Tetraspanins on the exosome surface mainly mediate cell adhesion and participate in maintaining the optimal conformation of immune proteins like MHC class II, thus playing an important role in antitumor immunity through exosomal targeting to DCs^[61,63-65]^.

Therefore, the effect of exosomes’ immune stimulation depends mainly on their antigen presentation, while the effect of inhibiting immunity mainly depends on exosome-carried biological content consisting of ligands, miRNAs, and proteins, which may inhibit the cytotoxic activity of the NK and CD8^+^ T cells or increase suppressive immune cells such as MDSCs, Treg cells, and M2 macrophages. Understanding the effect of TEXs in immune regulation will allow for better understanding of the clinical application of TEXs in cancer diagnosis and treatment.

## TEXS SERVE AS DIAGNOSTIC AND PROGNOSTIC BIOMARKERS IN LUNG CANCER

TEXs and their content in biofluids, which represent the content of parent cells, may serve as newly developed non-invasive biomarkers for diagnosis, prognosis, and monitoring the efficacy of treatment in lung cancer^[9-11]^.

Jakobsen *et al*.^[66]^ examined the potential of exosomal proteins as diagnostic markers in advanced NSCLC. The EV (extracellular vesicle) array showed that the expression levels of CD9, CD63, and CD81 were significantly high in cancerous patients. Likewise, according to the EV array, NYESO-1, EGFR, and PLAP showed a strong correlation with a poor survival in NSCLC^[11]^. In addition, SRGN, TPM3, THBS1, and HUWE1 may serve as biomarkers to distinguish lung adenocarcinoma subjects from controls^[67]^. Combination of carcinoembryonic antigen, exosomal alpha-2-HS-glycoprotein and extracellular matrix protein 1 (ECM1) could improve the diagnostic accuracy of NSCLC^[68]^. Gao *et al*.^[69]^ indicated that plasma exosomal total protein, Tim-3 and Galectin-9 were significantly increased in NSCLC, and were positively associated with larger tumor size, advanced TNM stage, and distant metastases. The higher level of leucinerich a-2-glycoprotein (LRG1) was detected in urinary exosomes and may be a non-invasive diagnostic biomarker of NSCLC in urine^[70]^.

Several studies have shown that exosomal miRNAs may serve as potential biomarkers for the early diagnosis of lung cancer. Rabinowits *et al*.^[71]^ suggested that exosomal miRNAs in NSCLC patients very closely resemble those in NSCLC tissue, indicating that such a liquid biopsy may obviate the need to obtain tumor tissues. Tumor-derived exosomal miRNAs, adenocarcinoma-specific miR-181-5p, miR-361-5p, miR-30a-3p, and miR-30e-3p, and squamous cell carcinoma-specific miR-10b-5p, miR-320b, and miR-15b-5p, were isolated from the plasma of early-stage NSCLC patients, which are able to discriminate between adenocarcinoma and squamous cell carcinoma, thereby serving as noninvasive biomarkers for early diagnosis of NSCLC^[72]^. Exosomal miR-1169 and miR-260 have also been identified as potential biomarkers that can distinguish between early-stage wild-type EGFR and mutant EGFR NSCLC^[73]^. Exosomal miR-20b-5p and miR-3187-5p were drastically reduced in early-stage NSCLC patients than those in healthy controls, showing both exosomal miRNAs were efficient diagnostic biomarkers for early-stage NSCLC^[74]^. Exosomal miR-126 has also been identified as a possible diagnostic biomarker for NSCLC progression^[75]^. In fact, exosomes exist extensively in body fluids other than blood, and increasing attention has been focused on diagnostic assays in pleural fluid. Tamiya *et al*.^[76]^ revealed that exosomal miR-182 and miR-210 were demonstrated to have a diagnostic potential for differentiating lung adenocarcinoma pleural effusion from benign pleural effusion. Additionally, exosomal miR-200 and mRNA transcript encoding lipocalin-2 from pleural effusions may be considered as diagnostic markers to discriminate lung adenocarcinoma patients from patients with benign inflammatory processes^[77]^.

Recently, exosomal RNAs have been reported to predict the prognosis of a variety of cancers including lung cancer. Dejima *et al*.^[78]^ showed that exosomal miR-21 and miR-4257 levels of the NSCLC patients were significantly upregulated during recurrence and can be used as recurrence-specific biomarkers. Additionally, another study reported that low exosomal let-7a-5p levels were significantly associated with a worse cancer-related survival rate in lung adenocarcinoma patients^[79]^. Luo *et al*.^[80]^ also demonstrated that serum exosomal miR-382 was considered as an independent prognostic biomarker for NSCLC. Zhang *et al*.^[81]^ suggested that exosomal lncRNA MALAT-1 was highly expressed in NSCLC patients and was positively associated with lymphatic metastasis and TNM stage, thereby indicating that exosomal MALAT-1 may be a non-invasive biomarker for diagnosis and prognosis of NSCLC. Moreover, tumor-derived exosomal eIF4E has the potential for use as a biomarker for survival prediction in NSCLC^[82]^.

Acquired resistance to general therapies, including chemotherapy, radiotherapy, immunotherapy, and targeted therapy, is a major challenge in the treatment of lung cancer. Nowadays, the use of exosomes as biomarkers for predicting therapeutic responses has gathered much attention. Exosomal hsa_circ_0014235 isolated from plasma promoted cisplatin chemoresistance and may serve as a promising biomarker for NSCLC treatment^[83]^. Exosomal miR-4443 might also promote cisplatin resistance of NSCLC by regulating FSP1-mediated ferroptosis^[84]^. In addition, Li *et al*.^[85]^ showed that plasma exosomal miR-92b-3p was significantly increased in chemoresistant small cell lung cancer patients and might serve as a potential dynamic biomarker for monitoring the drug resistance. Exosomal miR-29a-3p and miR-150-5p were identified as circulating biomarkers during thoracic radiation therapy for NSCLC and were correlated with delivered radiation therapy dose^[86]^. At present, precision medicine based on immunotherapy and targeted therapy has given new hope for lung cancer patients. The ensuing problems of drug resistance have gained much interest from the research community. For example, exosomal miR-323-3p, miR-1468-3p, miR-5189-5p, and miR-6513-5p were identified as potential biomarkers to discriminate between osimertinib-resistant and osimertinib-sensitive NSCLC patients^[87]^, and exosomal miR-210-3p secreted by osimertinib-resistant HCC827 and PC-9 cells was able to promote epithelial-mesenchymal transition and drug resistance in osimertinib-sensitive, EGFR mutant NSCLC cells^[88]^. Moreover, exosomal circRNA_102481 enhanced EGFR-TKIs resistance through the microRNA-30a-5p/ROR1 axis in NSCLC^[89]^. Peng *et al*.^[90]^ suggested that high exosomal miR320d, miR320c, and miR320b levels were corelated with poor response to anti-PD-1 treatment in patients with NSCLC, and exosomal miR-125b-5p was identified to be one potential target for anti-PD-1 treatment. In summary, exosomal RNAs in lung cancer could be used for monitoring therapy response/relapse to improve personalized therapy strategies.

## THE ROLE OF TEXS IN IMMUNOTHERAPY OF LUNG CANCER

### Effect of TEXs for resistance to immunotherapy

TEXs may suppress proliferation and differentiation of immune cells and have the ability to influence their biological function. TEXs carrying a variety of tumor associated antigens or immunoinhibitory mediators not only suppress antitumor functions of immune effector cells, but also appear to impede effective response to immunotherapy in cancer^[8]^. Tumor associated antigens on TEXs could efficiently bind antibodies produced against cancer cells and block the access of therapeutic antibodies to the cancer cells, leading to a decrease in effectiveness of cancer therapy^[91]^. Additionally, TEXs are able to inhibit antibody dependent cell-mediated cytotoxicity, which serves as a critical mechanism of therapeutic antitumor activity of anticancer antibodies^[91]^. During therapy, immune escape in NSCLC occurs through a multistep process that facilitates tumor growth and progression. Acquired tumor resistance to immunotherapy could be directly reflected in the production of TEXs^[92]^. Most importantly, Kim *et al*.^[93]^ showed that lung cancer cells increased their production of immunosuppressive exosomes during acquired resistance to anti-PDL1 immunotherapy.

### TEXs-based cancer vaccine and immunotherapy

TEXs are nanoscale membrane-derived vesicles that are thought to be important mediators of intercellular communication. Moreover, TEXs with distinct characteristics such as stability, permeability, biocompatibility, low immunogenicity, and low toxicity can efficiently deliver tumor antigens to DCs, thus they can be used as self-antigen carriers to stimulate immune response^[94-96]^. Increasing evidences have demonstrated that the activation and maturation of DCs by TEXs could enhance anti-tumor effects and may be applied for lung cancer immunotherapy. For example, TEXs from *CD40L*-gene modified 3LL lung tumor cells have the potent ability to activate DCs, resulting in significantly increased tumor antigen-specific CD4^+^ T cell proliferation and CD8^+^ T cell responses, revealing a powerful antitumor effect^[97]^. In addition, the exosomes derived from Rab27-overexpressing NSCLC cells also stimulated the proliferation and maturation of DCs effectively, promoted CD4^+^ T cell proliferation and elicited potent antitumor immune responses^[98]^. Multiple studies have already proved that TEXs which were used as tumor antigens source for DC vaccines, have greater efficacy and safety than conventional tumor cell lysates^[99-103]^. Wang *et al*.^[104]^ indicated that TEXs were more potent than tumor cell lysates to trigger DC-mediated immune responses and decrease Tregs, contributing to improving vaccine-elicited immunotherapy for lung cancer. Thus, DCs loaded with TEXs may be promising therapeutics without severe side effects and treatment resistance in clinical application^[105]^.

## CONCLUSION

TEXs are important mediators of intercellular communication and have been proven to play a key role in the TME. The biogenesis and secretion of TEXs have been widely reported. They carry a variety of cargoes and are involved in both immunosuppressive and immunostimulatory signaling pathways by delivering molecular signals to immune cells. The small size of TEXs and their contents render them highly interesting for biomedical applications, such as biomarker molecules and anticancer vaccines. Based on the data herein, we suggest that TEXs could be manipulated to provide clinical benefits and improve the clinical management of lung cancer.

## DECLARATIONS

### Copyright

© The Author(s) 2022.
